# Investigation on the Correlation between Biaxial Stretching Process and Macroscopic Properties of BOPA6 Film

**DOI:** 10.3390/polym16070961

**Published:** 2024-04-01

**Authors:** Bowen Li, Guangkai Liao, Yuankang Li, Haomin Yin, Lingna Cui, Kaikai Cao, Zhenyan Xie, Jiaxin Liu, Yuejun Liu

**Affiliations:** Key Laboratory of Advanced Packaging Materials and Technology of Hunan Province, Hunan University of Technology, Zhuzhou 412007, China; bowenli1125@163.com (B.L.); lyk05112020@163.com (Y.L.); 15773999509@163.com (H.Y.); lncui1102@126.com (L.C.); caokk@csrzic.com (K.C.); xzy045@foxmail.com (Z.X.); jiaxinl1010@163.com (J.L.)

**Keywords:** biaxial stretching, polyamide 6, film, rheology, crystallization, mechanical properties, barrier properties, optical performance

## Abstract

Biaxially oriented polyamide 6 (BOPA6) films were prepared by extrusion casting and biaxial stretching with polyamide 6. The effects of different biaxially oriented on the macroscopic properties of BOPA6 were investigated by characterizing the rheological, crystallization, optical, barrier and mechanical properties. The results show that the increase of stretching temperature leads to the diffusion and regular stacking rate of BOPA6 chain segments towards crystal nuclei increases, the relative crystallinity increases, reaching 27.87% at 180 °C, and the mechanical strength and optical performance decrease. Heat-induced crystallization promotes the transformation of β-crystals to α-crystals in BOPA6, resulting in a more perfect crystalline structure and enhancing oxygen barrier properties. BOPA6 chains are oriented, and strain-induced crystallization (SIC) occurs during the biaxial stretching. Further increasing the stretch ratio, the relative crystallinity increased to 30.34%. The machine direction (MD) and transverse direction (TD) tensile strength of BOPA6 (B-33) are nearly two times higher than the unstretched film, reaching 134.33 MPa and 155.28 MPa, respectively. In addition, the permeation decreases to 57.61 cc·mil/(m^2^ day), and the oxygen barrier performance has improved by nearly 30% compared to the sample B-22. BOPA6 has a high storage modulus at a high stretching rate (300%/s). Rapid chain relaxation would promote the molecular chain disorientation, destroy the entangled network of the molecular chain, and lead to a decrease in tensile strength, reducing to about 110 MPa.

## 1. Introduction

Biaxially stretched nylon 6 film (BOPA6) is a material produced by extrusion casting polyamide 6 chips and subsequently undergoing synchronous biaxial stretching [1]. It has excellent mechanical properties, high light transmittance, and excellent gas barrier properties and is widely used in food packaging, biomedicine, optical displays, and other fields [2,3,4]. Biaxial stretching refers to the simultaneous stretching of the polymer film in the machine direction (MD) and the transverse direction (TD), which can precisely control the microstructure of the polymer to promote the crystallization process, ensure the orderly arrangement of molecular chains and improve the physical and chemical stability of the film, it has become one of the main production methods of high-performance film [5,6,7]. As a typical semi-crystalline polymer, PA6 molecular chains will form partially ordered crystalline regions and partially amorphous regions during the biaxial stretching process, which will affect the macroscopic properties and properties of the material [8,9]. The biaxial stretching process (stretching temperature, stretching ratio, stretching rate) will directly affect the microstructure and macroscopic properties of BOPA6 [10]. At present, the investigation on BOPA6 mainly focuses on polycrystalline phenomena and crystal structure [11,12], and the structural evolution and macro property changes of BOPA6 under different bidirectional tensile conditions are not systematic and in-depth, which limits the performance optimization of BOPA6 films to a certain extent.

In recent years, researchers have performed extensive research on the structure and properties of BOPA6 film. Ziabicki [13] first proposed that the molecular chains of β-form in PA6 are arranged in parallel to each other to form a pseudo-hexagonal structure, and hydrogen bonds are randomly distributed on the chain axis. Zhang X et al. [14] studied the isothermal crystallization of PA6 by using an improved rapid scanning calorimeter. It was found that the melt-crystallized PA6 formed a mesophase *β* phase below 105 °C and a phase above 150 °C. The γ phase coexisted with the *β* phase or α phase in the temperature range of 105~150 °C. Despite the highly regular chain structure, low polydispersity, and the presence of hydrogen bonds strengthening the interactions between molecular chains, the crystallinity of PA6 is quite low, typically less than 35%. Chen J [15] stretched the PA6 sheet at 120 ° C to achieve a highly aligned crystal structure. The X-ray diffraction results showed that the crystal orientation of the precursor sheet was 42% higher than that of the sample, and the tensile stress induced γ-α phase transition and increased crystallinity. Al-Itry, R et al. [16] investigated the effects of biaxial stretching above the glass transition temperature on the structure and properties of PLA, PBAT, and their blends. Through dynamic mechanical and thermal analysis (DMTA), differential scanning calorimetry (DSC), and other characterization methods, it was found that strain-induced crystals (SIC) of PLA combined mesophase and α-form crystals, and the crystallization rate and stress increased. In addition, compared to uniaxial stretching, PLA, PBAT, and their blends exhibited improved toughness after biaxial stretching. Chen Q. et al. [17] studied the deformation behavior and structural changes of biaxially stretched polyethylene (BOPE) films during the stretching process and found that BOPE spherules were broken into small pieces after biaxially stretched, and the broken layers and newly formed crystals uniformly distributed on the MD-TD plane to form an isotropic fibrous network. Moreover, compared with the unstretched sample, the films could achieve up to about two times the tensile modulus and 4.5 times the tensile strength and also exhibit excellent optical properties. Shi et al. [18] successfully prepared highly oriented polyamide 6 (PA6) films by solid thermal stretching technology and studied the effect of orientation on its structure and thermal oxidation stability. The results demonstrate that the crystallinity and chain orientation of BOPA6 increase with higher stretching ratios, leading to a decrease in the degradation rate constant (k1); the oriented PA6 film exhibits higher activation energy (*E*_a_) and improved thermal oxidation stability. In addition, the oxygen barrier performance is enhanced due to the increased crystallinity and formation of a stable and dense α-crystalline structure induced by orientation. Lin et al. [19] used small-angle X-ray scattering (SAXS) and scanning electron microscopy (SEM) to investigate the effect of tensile rate on the structure and mechanics of hard elastic polyethylene films and found that stress-induced microphase separation of amorphous phases triggered the yield behavior, and the yield behavior distribution became more uniform with the increase of tensile rate. The final macroscopic properties of BOPA6 are closely related to the biaxial stretching process (stretching temperature, stretching ratio, stretching rate). Therefore, the correlation between the biaxial stretching process and the macroscopic properties of BOPA6 film is of great significance for the processing of biaxially stretched PA6 film.

In this paper, the relationship of different bidirectional drawing processes to the macroscopic properties of PA6 films has been systematically studied. Firstly, the rheological properties of PA6 were tested, and the extrusion casting conditions and biaxial stretching process were designed. Then, the PA6 casting film was prepared by melting, extrusion, casting, winding, and other processes. The BOPA6 film was prepared by biaxial synchronous stretching of the casting film under different biaxial stretching processes (different stretching temperatures, different stretching ratios, different stretching rates) using a biaxial stretching test machine. In order to investigate the crystallization, barrier, optical, mechanical, and other properties of BOPA6 films with different biaxial stretching conditions, various characterizations of BOPA6 films were carried out to establish a bridge between BOPA6 film processing, microstructure, and macroscopic properties, which provided some theoretical guidance for optimizing biaxial stretching process and BOPA6 structural properties.

## 2. Materials and Methods

### 2.1. Materials

Raw material polyamide 6 was purchased from Hunan Yuehua Chemical Co., Ltd. (Hengyang, China) with a purity of 99.9%. Density: 1.158 g/cm^3^, melt index: 38 g/10 min, melting temperature: 220–225 °C.

### 2.2. Preparation of BOPA6 Film

Due to the hygroscopic nature of PA6, the raw material slices were first dried in an electric constant temperature blast drying oven (HGZF-101-2, Shanghai Yuejin Medical Instrument Co., Ltd., Shanghai, China) at 80 °C for 8 h to remove surface moisture. Then, the material was placed in a vacuum drying oven (DFZ-6053, Shanghai Yiheng Scientific Instrument Co., Ltd., Shanghai, China) to dry at 120 °C for 12 h and observe whether the vacuum meter value changed every 2 h, and if significant changes were detected, the vacuum pumping operation was repeated.

**Figure 1:**
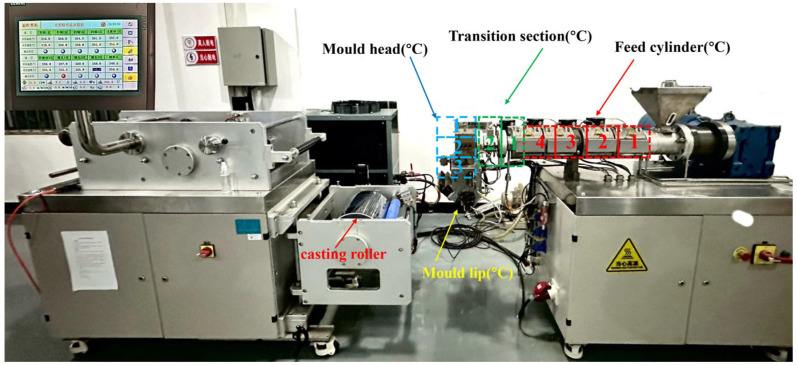
Extrusion casting machine temperature zone distribution.

Polyamide 6 (PA6) pellets were melted and extruded by a single screw extrusion casting machine (FDHU35, Guangzhou General Experimental Analysis Instrument Co., Ltd., Guangzhou, China) to prepare uniformly thick and impurity-free cast film. In order to ensure the extrusion quality, the extrusion equipment is washed first with the cleaning machine material. In the process of extrusion melting, PA6 granules pass through four temperature zones in the extrusion casting machine: the feed cylinder, transition section, mold head, and mold lip, as shown in Figure 1.

The parameters of the extruder temperature zone are shown in Table 1. The temperature of the Feed cylinder zone 1 is set near the melting point to prevent rapid incomplete melting from blocking the channel; the temperatures in the subsequent zones are kept around 30 °C higher than the melting point of PA6 to ensure smooth melting and uniform flow of the raw material.

The lower temperature of the mold lip is intended to control the cooling rate and ensure uniformity of the product. The screw speed of the extruder is 30 r/min. The PA6 melt was extruded and cooled on a casting roller with a set temperature of 30 °C, and then the casting film with a thickness of 0.250–0.300 mm was obtained by traction and winding.

**Figure 2:**
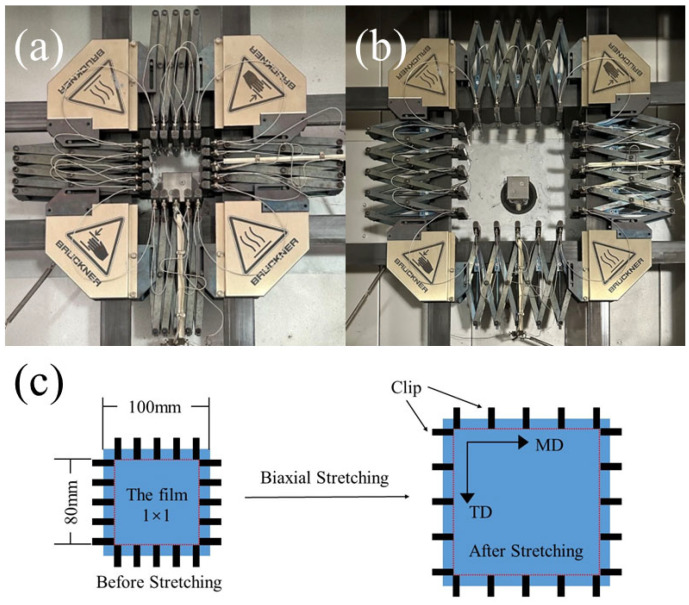
(**a**) Laboratory-produced 100 mm × 100 mm PA6 cast film, (**b**) biaxially stretched BOPA6 film, (**c**) schematic diagram of the biaxial stretching process.

The prepared cast film was cut into 100 mm × 100 mm samples, as shown in Figure 2a. BOPA6 film was prepared by biaxial synchronous stretching of PA6 cast film with different stretching processes (stretching temperature, stretching ratio, stretching rate) by biaxial stretching test machine (KARO-5, Bruckner, Siegsdorf, Germany), as shown in Figure 2b, where the casting direction is machine direction (MD), and the vertical casting direction is transverse direction (TD). In the experimental process, five different stretching temperatures (70 °C, 90 °C, 120 °C, 160 °C, 180 °C) are set as independent variables for sample W. Due to restricted molecular chain movement near the glass transition temperature, it is difficult to achieve a large stretching ratio. Therefore, under this experimental condition, the stretching ratio is set to 2 × 2, and the stretching rate is 100%/s. Investigate the effect of different stretching ratios on material properties; five different stretching ratios (1 × 1, 1.5 × 1.5, 2 × 2, 2.5 × 2.5, 3 × 3) are set as independent variables for sample B, the stretching temperature is 160 °C, and the stretching rate is 100%/s. Finally, five different stretching rates (25%/s, 50%/s, 100%/s, 200%/s, 300%/s) are set at a stretching temperature of 160 °C and a stretching ratio of 2.5 × 2.5 for sample S. In order to maintain a single variable, the heat setting temperature of all samples is 30 °C higher than the stretching temperature, and the biaxial stretching process is shown in Table 2.

The PA6 film was preheated in the stretching chamber for 30 s to reach the stretching temperature before biaxial stretching to reach the stretching temperature and then moved into the heat-setting chamber for 60 s after biaxial stretching. The biaxial tensile test machine can collect the experimental data of load, displacement and time during the stretching process. In order to ensure the repeatability of the experimental data, five repeated experiments were carried out under different stretching processes. The Schematic diagram of the biaxial stretching process is shown in Figure 2c.

### 2.3. Testing and Characterization

Rheological performance test: Due to the hygroscopic nature of PA6, the specimens should be dried in advance before testing. PA6 samples were tested by a rotary rheometer (AR200ex, TA, Novi, MI, USA) equipped with a conical fixture, and the test was carried out in a nitrogen atmosphere to prevent sample degradation. Using dynamic frequency scanning, the strain is set to 1%, the angular frequency is 0.1–500 rad/s, and the test temperature is 235 °C, 245 °C, and 255 °C.

Differential Scanning Calorimetry (DSC) testing: BOPA6 films were tested via experiments under a nitrogen atmosphere by differential scanning calorimetry instrument (Q20, TA, New Castle, DE, USA). The sample mass was 4–6 mg, and the temperature was increased from 25 °C to 300 °C at a heating rate of 10 °C/min. The melting temperature (*T_m_*) and melting enthalpy (Δ*H_m_*) were measured. Crystallinity (%) [20] can be calculated using Equation (1):

(1) $$K_{c}=\frac{\Delta H_{m}}{\Delta H_{f}^{*}}$$

where *K_c_* is crystallinity, Δ$H_{f}^{*}$ was the theoretical melting enthalpy change value of the material when PA6 is completely crystallized, which is 230 J/g [21].

X-ray diffraction (XRD) testing: BOPA6 films under different biaxial stretching processes were tested using an X-ray diffractometer (Smart-lab SE, Rigaku, Tokyo, Japan). The test environment is a reflection mode and Cu Kα radiation (λ = 0.154 nm), the operating voltage and current are 40 kV and 30 mA, respectively, the test range is 5~40°, and the scanning rate is 2°/min. The average grain size of crystal particles can be determined by the Debye–Scherrer equation [22] (Equation (2)) analysis:

(2) $$D=\frac{k\lambda }{\beta \cos\theta }$$

where *D* represents the grain size (average crystal grain size), *k* is the Scherrer constant, typically taken as 0.9, *λ* is the X-ray wavelength, *β* represents the peak width of the X-ray diffraction, *θ* represents the diffraction angle.

Miller indices [23] are a method for representing crystal plane direction and can be determined using Bragg’s law [24] (Equation (3)):

(3) nλ=2dsinθ

where *n* represents the diffraction order, *λ* is the wavelength of the incident radiation, *θ* is the diffraction angle, and *d* is the spacing between crystal planes.

Mechanical properties test: The mechanical properties of BOPA6 film in MD direction and TD direction were tested by film electronic universal tensile testing machine (502B-EX, Shenzhen Wance Experimental Equipment Co., Ltd., Shenzhen, China). Before conducting the test, the thickness of the sample should be measured. The testing conditions include a temperature of 25 °C, humidity of 68 %RH, and tensile speed of 50 mm/min. Specimens of the test specimen are provided in Table 3. Each group of samples was subjected to 5 repeated experiments to ensure the availability of the results, and the tensile strength and elongation at break were obtained by force-displacement data.

Oxygen transmission rate test: Using the oxygen permeability tester (OX-TRAN 2/21, MOCON, Brooklyn Park, MN, USA), oxygen barrier properties of BOPA6 films with different biaxial stretching processes were tested by equal pressure methods. The sample size is 5 cm^2^, the experimental environment temperature is 25 °C, and the humidity is 50–70 %RH. The pressure of the permeating gas needs to be maintained constant, using equal pressure conditions with a pressure difference of less than 1% on both sides. The thickness of the sample should be measured before each test, and the same set of experiments should be repeated more than twice to ensure data availability.

Optical test: BOPA6 films were tested by transmittance/haze meter (WGT-S, Jinan Languang Mechanical and Electrical Technology Co., Ltd., Jinan, China). Prior to the experiment, the film should be cut into square pieces measuring 30 mm × 30 mm and placed on a transparent window. Each set of experiments should be repeated three times to ensure data reliability.

Dynamic mechanical analysis (DMA) test: The MD direction of BOPA6 films was tested by a dynamic thermomechanical analyzer (DMA242E, NETZSCH, Selb, Germany) equipped with a stretching fixture. (1) Standard temperature scanning test: Single frequency temperature scanning was performed on BOPA6 films with different biaxial stretching processes; the temperature range was 30–160 °C, the heating rate was 3 °C/min, the frequency was 1 Hz, and the absolute target amplitude was 20 μm to control the test conditions to ensure the accuracy and repeatability of the results. (2) Multi-frequency temperature scanning: The loading frequencies were 1, 2, 2.5, 3.33, 5, 10, 16.66, 20, 33.33 and 50 Hz, and the heating rate was increased from 30 °C to 150 °C at 3 °C/min. In order to ensure that the test results are in the linear viscoelastic range of the material, the strain amplitude of the material under dynamic loading is 0.1%, and the pre-strain is 0.4%. The activation energy of BOPA6 can be obtained by Arrhenius law [25] (Equation (4)):

(4) $$K=Ae^{-E_{a}/RT}$$

where *K* is the physical quantity of the system, *A* is a constant, *E_a_* is the activation energy, *T* is the thermodynamic temperature, and *R* is the molar gas constant. Determine the glass transition temperature (*T_g_*) at different loading frequencies; the peak of the loss factor is defined as *T_g_* in the DMA curve [26]. Taking the logarithm of Equation (4), the activation energy formula calculated by Arrhenius is as follows:

(5) $$\ln(k)=\ln(A)-(E_{a}/R)\times (1/T_{g})$$

## 3. Results and Discussion

### 3.1. Biaxial Stretching Data Analysis

**Figure 3:**
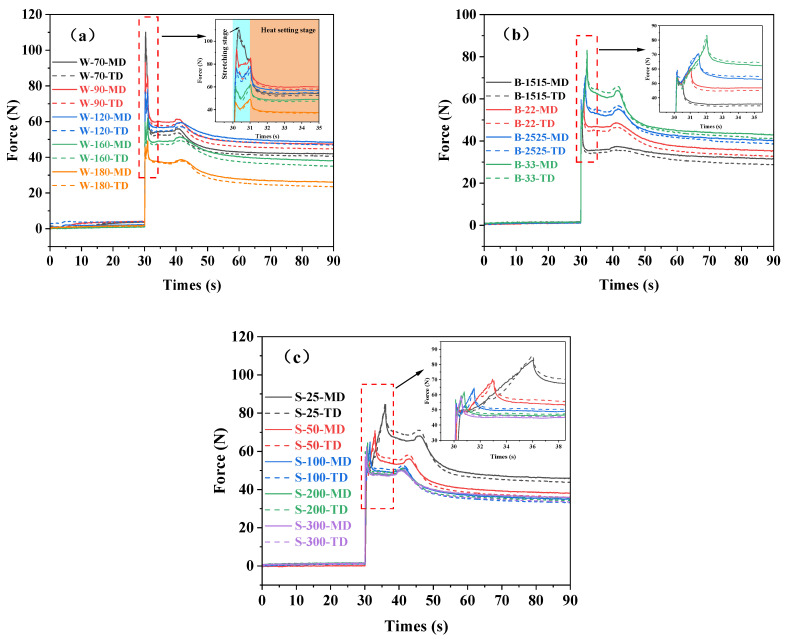
Force–time curves of BOPA6 film under different biaxial stretching processes: (**a**) different stretching temperatures, (**b**) different stretching ratios, (**c**) different stretching rates.

Biaxial stretching of PA6 films with different processes was carried out above the glass transition temperature. Figure 3 shows the curves of force and time during biaxial stretching under different stretching processes. It can be seen from the force–time curves of BOPA6 films at different stretching temperatures (Figure 3a):

(1) With the increase in temperature, the force required for the casting film during the biaxial stretching process decreases; the force required for the biaxial stretching is 45 N at 180 °C, which is 55% lower than that at 70 °C. This indicates that high-temperature conditions will promote the movement of polymer chains and improve the flexibility and ductility of the film, thereby reducing the force required for the stretching process. At high temperatures, the molecular slip of the BOPA6 internal structure is more active under the action of external force, which makes the material more prone to deformation [27,28]. During stretching and thermal shaping processes, the force in the machine direction (MD) is consistently higher than that in the transverse direction (TD) because the PA6 film exhibits significant anisotropy during the extrusion process, resulting in smoother stress transmission in the MD direction during biaxial stretching. In the heat setting stage (post 40 s), the force was increased from 70 °C to 120 °C and then decreased from 120 °C to 180 °C. The reason is that although the setting temperature of the heat setting chamber is 30 °C higher than that of the stretching chamber, due to the different stretching temperatures, BOPA6 experienced significant temperature variations when exposed to different temperature environments during the transfer process, resulting in differences in the molecular structure and state of the samples, such as shape memory effect, crystallization behavior and molecular entanglement effect, which in turn affect the order of force in the heat setting stage;
(2) The force–time curves in the biaxial stretching process under different stretching ratios are shown in Figure 3b, indicating that the force required for initial deformation is basically the same, approximately 60 N. At the same stretching temperature and rate, the force required to complete the stretching process with a stretching ratio of 1.5 × 1.5 is 50 N, which is less than the force needed for the initial deformation, and the stretching completion time is 0.5 s. When the stretching ratio is 3 × 3, the force required to complete the stretching is 85 N, and the stretching completion time is 2 s. This is because the molecular structure of the material has not been greatly stretched and deformed when the stretching ratio is small. As the stretching ratio increases, the strain difference of BOPA6 becomes larger, and the obvious change of the molecular structure needs to extend a longer distance to maintain the overall structure, thus increasing the interaction force between the molecules, resulting in stress concentration. Macroscopically, the force required for biaxial stretching is increased. When the stretching ratio is small (1.5 × 1.5, 2 × 2), the force in the MD direction is always greater than that in the TD direction during the stretching stage and the heat setting stage. However, it can be found that the force in the MD direction will be less than the force in the TD direction at a large stretching ratio (2.5 × 2.5, 3 × 3) after the stretching is completed after the BOPA6 is moved into the heat setting chamber (post 40 s), the same phenomenon as observed with a small stretching ratio occurs. This is because as the stretching ratio increases, the concentration of stress in the TD direction increases. During the heat setting stage, due to the molecules having enough time to rearrange, the stress difference between the MD direction and the TD direction is twisted so that the force in the MD direction is greater than that in the TD direction;
(3) The stretching temperature of 160 °C and the stretching ratio of 2.5 × 2.5 were selected in this group of experimental conditions because the film structure was more uniform at a high stretching ratio and temperature. The force–time curves of samples at different stretching rates during the biaxial stretching process are shown in Figure 3c. It can be observed that when the stretching rate increases from 25%/s to 50%/s, the force decreases from 85 N to 71 N, and the stretching time is reduced to 3 s. At higher stretching rates (100%/s, 200%/s, 300%/s), the force required for biaxial stretching is approximately the same, about 64 N, which is 24.7% lower than that at 25%/s. This indicates that higher stretching rates are beneficial in reducing the force requirement during the biaxial stretching process and shortening the stretching time. In addition, it can be observed that after stretching under these experimental conditions, the force in the TD is greater than that in the MD. However, after BOPA6 enters the heat-setting chamber, the force becomes greater in the MD direction than in the TD direction, which is consistent with our previous analysis under high stretching ratios.

### 3.2. Rheological Properties

**Figure 4:**
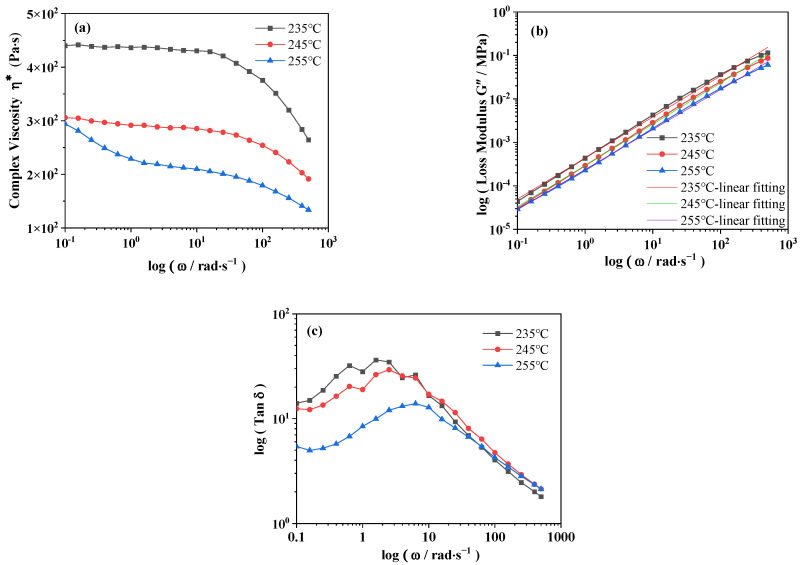
PA6 rheological curve: (**a**) the logarithmic relationship curve of complex viscosity (η*) and angular velocity (*ω*) at different temperatures, (**b**) the logarithmic relationship curve of loss modulus (*G*″) and angular velocity (*ω*) at different temperatures, (**c**) the logarithmic relationship curve of loss factor (Tan δ) and angular velocity (*ω*).

The melting point of PA6 is 220–225 °C, and the rheological test is carried out at 15–35 °C higher than the melting point to explore the rheological properties of PA6 under dynamic frequency scanning. It can be seen from Figure 4a that the complex viscosity (η*) of PA6 decreases with the increase of logarithmic angular frequency (*ω*) at the same temperature, showing the shear thinning of the melt, which is consistent with the typical characteristics of the polymer. The main reason for this phenomenon is that the conformation of the molecular chain changes, and the chain segment orientation of BOPA6 occurs, the macromolecules are easier to move, resulting in a decrease in melt viscosity. In addition, there are many entanglement points inside BOPA6. These entanglement points are destroyed when the shear rate increases and the entanglement density decreases, resulting in a decrease in viscosity. At the same frequency, the complex viscosity of PA6 decreases with the increase of temperature because the high temperature leads to more intense and free molecular motion, and the molecules have higher kinetic energy at high temperature, which makes it easier to overcome the intermolecular force, thus reducing the viscosity of the material.

Figure 4b is the logarithmic relationship curve of loss modulus (*G*″) and *ω* of PA6 at different temperatures. According to the power law, the slope at each temperature is obtained by linear fitting, which is the non-Newtonian index (N), which can be used to characterize the dependence of shear viscosity on shear rate (or frequency) at high temperatures. The non-Newtonian index of PA6 at different temperatures were N_235_ = 0.949, N_245_ = 0.943, N_255_ = 0.918, respectively. It can be observed that the non-Newtonian index decreases with increasing temperature, indicating improved flow behavior of PA6; the sensitivity to frequency decreases at high temperatures, leading to improved film-forming properties during processing. At the same temperature, the PA6 molecular chain is not fully arranged due to the high shear rate, and the energy is dissipated in the form of frictional heat, which leads to the increase of loss modulus.

Figure 4c shows the logarithmic relationship between the loss factor (Tan δ) and *ω* at different temperatures. It can be found that compared to the Tan δ curve at 235 °C, the peak value of Tan δ at 245 °C exhibits a slight decrease and shifts towards higher temperatures. As the temperature increases, the interaction force between molecular chains is weakened, and the molecular chain motion is more active and frequent, which makes the viscoelastic response of the material shift to high frequency and reduces the energy dissipation corresponding to the molecular motion. However, at 255 °C, the Tan δ peak shows a significant shift and decrease compared to 235 °C and 245 °C. This phenomenon can be explained as the reduction of entanglement of macromolecular chains and the more active chain motion; the material is more sensitive to changes in temperature and frequency, molecular chain breakage results in a more pronounced shear thinning phenomenon, and even small changes may cause significant changes in rheological behavior. According to the significant temperature and frequency dependence of PA6, increasing the temperature and shear rate during processing, the activity of the PA6 molecular chain increases, the energy barrier to be overcome decreases, and the sensitivity to temperature decreases, which is conducive to improving the uniformity of the film during extrusion casting.

### 3.3. Crystallization Properties of BOPA6

**Figure 5:**
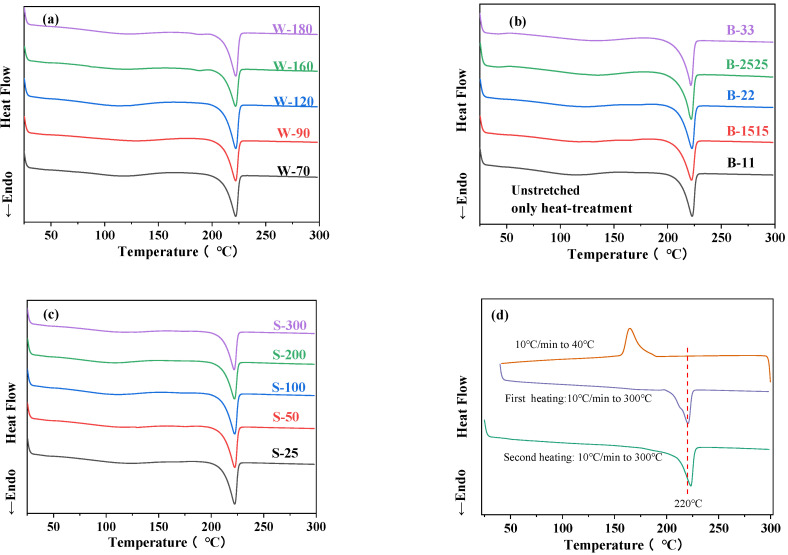
Different biaxial stretching process BOPA6 one-time heating and un-stretched two times heating DSC curve: (**a**) different stretching temperature, (**b**) different stretching ratio, (**c**) different stretching rate, (**d**) unstretched two times heating.

Figure 5 is the DSC heating curve of BOPA6 film samples under unstretched different and biaxial stretching processes to study the influence of different biaxial stretching processes on crystallinity. The data on thermal properties and crystallization properties are shown in Table 4.

Figure 5a is the DSC heating curve of BOPA6 film at different stretching temperatures. Combined with Table 4, it can be seen that after undergoing biaxial stretching at different temperatures, the melting point (*T_m_*) of BOPA6 did not exhibit significant changes, about 222 °C, while the melting enthalpy and relative crystallinity changed in different degrees. Under the same stretching ratio and stretching rate, the melting enthalpy and relative crystallinity of BOPA6 increased with the improvement of stretching temperature. Compared with 70 °C, the melting enthalpy of BOPA6 at 180 °C increased by 3.23%, and the relative crystallinity increased by 2.39%. The reason is that during the stretching process, heat-induced crystallization at higher temperatures generates α crystals, thereby forming a more complete crystal structure and increasing the melting enthalpy and relative crystallinity.

Figure 5b is the DSC heating curve of BOPA6 film samples under different stretching ratios to study the crystallization behavior of the film after biaxial stretching. For the unstretched and heat-treated PA6 cast film sample (B-11), the secondary heating DSC curve (Figure 5d) shows that the melting point is about 223 °C. However, the melting point of BOPA6 decreased significantly from 223 °C to 221 °C when the tensile ratio was 3 × 3 after biaxial stretching with different stretching ratios, which was beneficial for material processing and could be carried out at lower temperatures. The unstretched BOPA6 sample undergoes a crystalline phase transition near 180 °C, and the melting enthalpy changes significantly after biaxial stretching. When the stretching ratio is 3 × 3, the melting enthalpy is 69.785 J/g, which increased by 32.39% compared with the unstretched sample. This indicated that strain-induced crystallization (SIC) occurs during the stretching process [29], and the orientation of the amorphous chain forms the crystal nucleus. With the increase of the stretching ratio, the BOPA6 molecular chains are more orderly arranged, the crystallinity is enhanced, and the amorphous region is reduced, thereby increasing the melting enthalpy.

It can be seen from Figure 5c that the melting enthalpy and relative crystallinity decrease significantly with the increase of tensile rate. When the stretching rate is 300 %, the melting enthalpy decreases to 49.919 J/g, which is 21.68% lower than the stretching rate of 25%/s. This is because, at a high stretching rate, BOPA6 molecular chains do not have enough time to adjust their alignment and orientation, thus slowing down the occurrence of crystallization in the amorphous region. However, the effect of different stretching rates on the melting point of BOPA6 is not obvious at about 221 °C.

**Figure 6:**
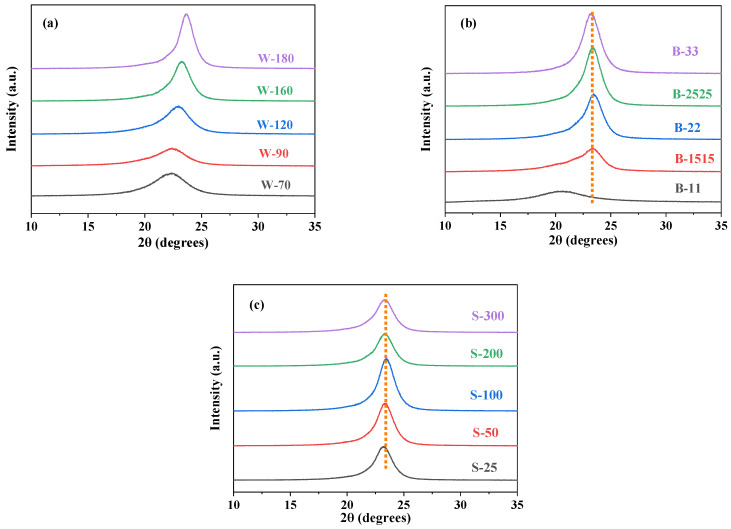
XRD patterns of BOPA6 films with different biaxial stretching processes: (**a**) different stretching temperatures, (**b**) different stretching ratios, (**c**) different stretching rates.

XRD data in Figure 6a show that under different biaxial stretching processes, BOPA6 exhibits a unique wide diffraction peak between 2θ = 20 ° and 2θ = 25 °, indicating its main crystalline phase is the unstable β-crystal form [21]. At high temperatures, grains may undergo recrystallization or grain growth, the full width at half maximum (FWHM) of the X-ray diffraction peak of BOPA6 decreases (Table 4), and the grain size increases from 22 Å at a stretching temperature of 70 °C to 35 Å at a stretching temperature of 180 °C, resulting in an increase in the intensity of the X-ray diffraction peak. This indicates that with the increase in stretching temperature, the extent of transformation from the β-crystal form to the α-crystal form increases, resulting in a higher proportion of the α-crystal form and a more perfect crystal structure. The slight rightward shift of the diffraction angle may be due to subtle changes in lattice parameters and crystal structure.

XRD data of BOPA6 with different tensile ratios are shown in Figure 6b. BOPA6 film with a stretching ratio of 1 × 1 has a deviated wide diffraction peak at about 2θ = 21°, which is due to the relatively large number of amorphous regions in the sample or the presence of a certain number of other phases in the sample. With the increase in stretching ratio, the XRD peak changed from a broad diffraction peak to a strong diffraction peak at 2θ = 24°, which corresponded to the α phase crystal form of PA6. At this time, the BOPA6 molecular chains tended to be arranged in an orderly manner under the action of stretching, which was a typical sign of the change of crystal structure induced by stretching, indicating that the stretching process increased the content and crystallinity of the α-crystal form. This indicates that stretching can improve the crystallinity of BOPA6 film, which is consistent with the analysis of front DSC. The grain size reached its maximum value at a stretching ratio of 2.5 × 2.5, which was 37 Å, indicating that stretching can promote the crystallization of BOPA6, but excessive stretching ratios can lead to a decrease in grain size.

Figure 6c shows the XRD curve at different stretching rates, and it can be observed that the full width at half maximum (FWHM) of the diffraction peaks after stretching has little change, about 2.2, indicating that the crystal structure has not changed significantly. However, the grain size reached its maximum value of 39 Å at a stretching rate of 100 %/s, indicating that there is an optimal stretching rate that promotes grain growth up to a certain extent, and then excessive stretching beyond this optimal rate can lead to a decrease in grain size. This is because the crystals in the BOPA6 film are influenced by high strain rates, which lead to an increase in the degree of crystal defects and disorder.

### 3.4. Mechanical Properties of BOPA6

**Figure 7:**
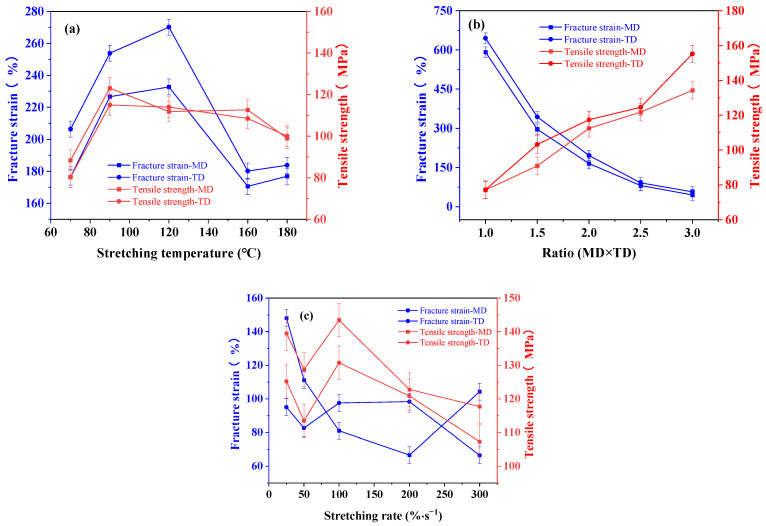
Failure strain and tensile strength of BOPA6 film in MD and TD directions with different biaxial stretching processes: (**a**) different stretching temperatures, (**b**) different stretching ratios, (**c**) different stretching rates.

Figure 7a shows the mechanical properties of BOPA6 film in MD and TD directions at different biaxial stretching temperatures.

With the increase in stretching temperature, the fracture strain of MD and TD initially increased and then decreased from 120 °C to 180 °C, reaching peak values at 120 °C; the tensile strength of MD and TD were 232% and 270%, respectively, indicating that at this temperature, the material had good toughness and could withstand large pre-fracture deformation. The fracture strain in the TD direction is consistently greater than that in the MD direction at each temperature, indicating that the material exhibits greater flexibility and ductility in the TD direction. The tensile strength in the MD and TD directions does not show significant differences at each temperature, reaching its maximum value at 90 °C, which is a 30 MPa increase compared to the temperature of 70 °C. At 90 °C and above, despite further temperature increases, the tensile strength shows little variation. This may be within this temperature range; the molecular structure of BOPA6 reaches a relatively stable state, resulting in the tensile strength remaining relatively constant.

Figure 7b shows the mechanical properties of BOPA6 film in MD and TD directions under different biaxial stretching ratios. Whether in the MD direction or the TD direction, the fracture strain of the unstretched sample (B-11) decreased from 600% to 75% under the condition of a 3 × 3 stretching ratio, showing a significant downward trend, indicating that the ductility of BOPA6 decreased, while the tensile strength of BOPA6 increased. When the tensile ratio was 3 × 3, it reached about 150 MPa, which was twice the tensile strength of the unstretched sample. This phenomenon can be explained as strain-induced crystallization occurs during the biaxial stretching process, which increases the degree of molecular chain orientation and relative crystallinity. The tensile strength increases with the increase of crystallinity, but the presence of the crystalline regions will reduce the fracture strain, resulting in BOPA6 which is harder but more brittle. In addition, due to the different orientations and arrangement of the BOPA6 molecular chain in MD and TD direction during the processing and preparation process, the molecular structure in TD direction has less orientation and better ductility, so the fracture strain and tensile strength in TD direction are always greater than in MD direction.

Figure 7c shows the mechanical properties of BOPA6 film in MD and TD directions at different biaxial stretching rates. The fracture strain in the MD direction decreases with increasing stretching rate, dropping from 148.243% at 25%/s to 66.622% at 200%/s, while the change in the TD direction is not significant. The tensile strength in the MD direction is consistently higher than in the TD direction, but both show an overall decreasing trend. This phenomenon can be explained by the different orientations during the preparation process and the reduced crystallinity due to inadequate orientation of the BOPA6 molecular chains at high stretching rates.

### 3.5. BOPA6 Oxygen Barrier Properties

BOPA6 film is used in the field of anti-oxidation packaging, such as food packaging, and oxygen barrier performance is very important. Table 5 shows the oxygen barrier properties of BOPA6 films under different biaxial stretching processes, including Transmission Rate and Permeation. The results show that as the stretching temperature increases, the Permeation reaches 66.273 cc·mil/(m^2^·day) at a stretching temperature of 180 °C, which is a decrease of 10.43% compared to sample W-140. This can be explained by the fact that at higher stretching temperatures, the enlargement of grain size and more orderly molecular arrangement reduce the chances of gas molecules passing through gaps, thereby enhancing the oxygen barrier properties of BOPA6.

The low transmission rate and high permeation of the B-22 sample indicate that BOPA6 has a certain degree of crystallization, but there are more amorphous regions at the same time, which makes it easier for gas to pass through the film. The permeation decreased by 27% from 79.377 cc·mil/(m^2^·day) of sample B-22 to 57.606 cc·mil/(m^2^·day) of sample B-33. It can be explained that the SIC occurs during the biaxial stretching process, resulting in the BOPA6 film reaching a better degree of crystallization and morphology with the increase of the stretching ratio, which leads to the improvement of the barrier performance. However, the sample of B-33 shows a moderate permeation; an excessive stretching ratio leads to a reduction in grain size, resulting in more amorphous regions being exposed to gas molecules, which contributes to gas permeability.

The sample S-100 has the lowest transmission rate of 22.662 cc/(m^2^·day) and permeation of 47.286 cc·mil/(m^2^·day), indicating that stretching at a lower rate can promote better molecular chain arrangement and crystallinity, resulting in higher oxygen barrier properties. As the stretching rate increases, the oxygen barrier properties of the BOPA6 film decrease. The reason is that the thermal and mechanical stress caused by the faster stretching rate makes it more difficult for the BOPA6 molecular chain to arrange in an orderly manner into a crystalline region, resulting in more amorphous regions so that the gas molecules can easily pass through the relevant. In addition, a high stretching rate will cause material damage or local stress concentration, affecting the overall performance of the film. As a result, the Transmission Rate exhibits a similar trend to that of different stretching temperatures and stretching ratios.

### 3.6. Optical Properties of BOPA6

**Figure 8:**
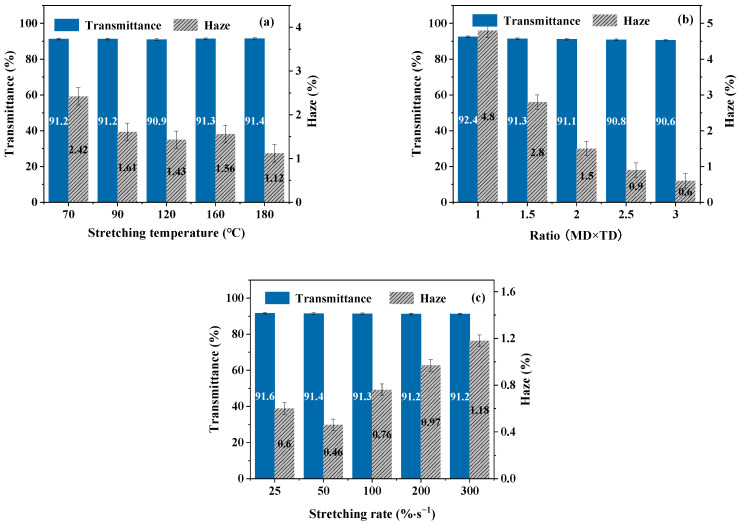
Transmittance and haze of BOPA6 films with different biaxial stretching processes: (**a**) different stretching temperatures, (**b**) different stretching ratios, (**c**) different stretching rates.

The optical properties of BOPA6 film have good application value in packaging and other fields, and the optical data are recorded in Figure 8.

The results show that the transmittance of BOPA6 film with different biaxial stretching processes is similar, which is above 90%. At a stretching temperature of 70 °C, the haze of BOPA6 is highest at 2.42%. However, at temperatures of 90 °C and above, there is a slight decrease in haze, but the difference is not significant (Figure 8a). This phenomenon can be explained by the significant influence of temperature on the internal structure of BOPA6 near the glass transition temperature, resulting in significant differences in haze. At high temperatures, the molecular chain activity increases, and the crystallinity of BOPA6 increases, which can reduce the disordered region in the material, thereby reducing the scattering of light through the film and ultimately reducing haze.

With the increase of the stretching ratio, the haze of BOPA6 decreased significantly (Figure 8b). When the stretch ratio is 3 × 3, the haze decreases by 4.2% compared to the unstretched sample (B-11). This can be explained by the fact that an excessively high stretching ratio (3 × 3) results in smaller grain size, and the molecular chains become more orderly oriented after experiencing significant tensile strain, leading to a more uniform and ordered internal structure that reduces light scattering in the film.

The optical properties of BOPA6 with different stretching ratios are shown in (Figure 8c). When the stretching rate increased from 25%/s to 50%/s, the haze of BOPA6 decreased from 0.6 to 0.46. However, with a further increase in the stretching rate, the haze rises significantly, reaching 1.8% at a stretching rate of 300%/s. This could be attributed to the molecular chain not having enough time to arrange and crystallize during the stretching process, and the internal structure changes unevenly.

### 3.7. Dynamic Mechanical Properties of BOPA6

**Figure 9:**
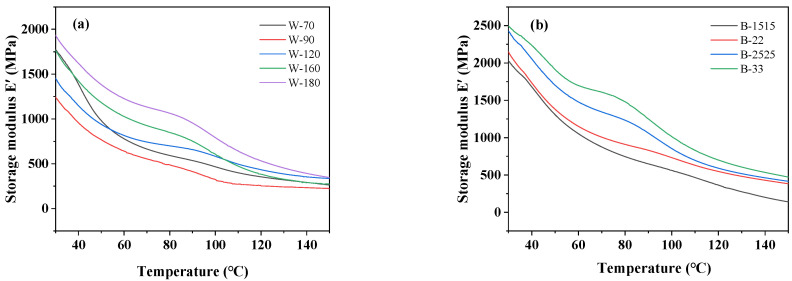

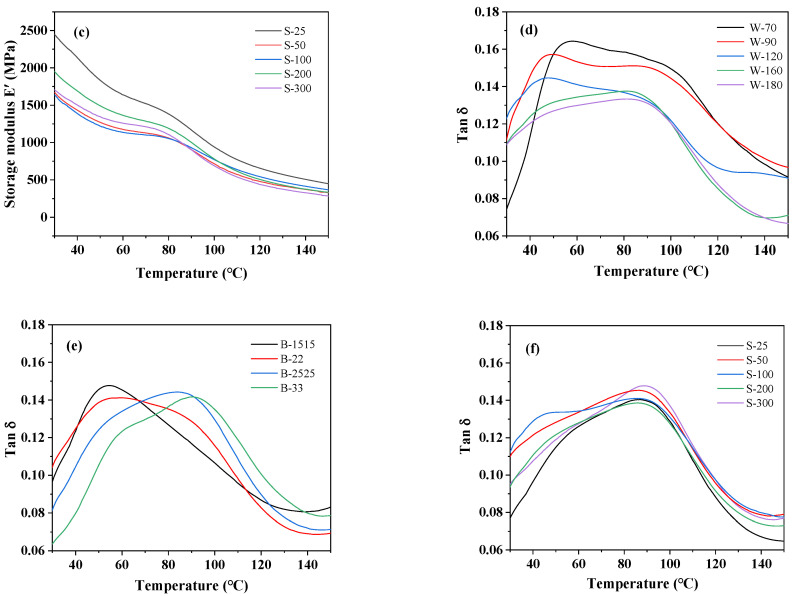
DMA curves of storage modulus (E′) and loss factor (tan δ) of BOPA6 films with different biaxial stretching processes changing with temperature: (**a**) storage modulus at different stretching temperatures, (**b**) storage modulus at different stretching ratios, (**c**) storage modulus at different stretching rates, (**d**) loss factor at different temperatures, (**e**) loss factor at different stretching ratios, (**f**) loss factor at different stretching rates.

The dynamic mechanical analysis of BOPA6 films with different biaxial stretching processes was carried out to reveal the correlation between microstructure and macroscopic properties. Figure 9 shows the DMA curves of storage modulus (E′) and loss factor (Tan δ) of BOPA6 films with different biaxial stretching processes. The results show the following:

The storage modulus curve of BOPA6 film shows a significant shift at different stretching temperatures, and the shift range is about 500 MPa from 90 °C to 180 °C (Figure 9a,d). It can be explained that within a temperature range above the glass transition temperature and below the melting point, the mobility of the BOPA6 molecular chain increases with temperature under external force, forming a more orderly arrangement structure, thereby improving the storage modulus. The decrease of storage modulus in the temperature range of 70 °C to 90 °C is due to the transition of PA6 from a glassy to rubbery state in the glass transition zone, that is, the α-transition process [30], which results in insufficient stiffness of BOPA6. The loss factor of BOPA6 decreases with the increase in stretching temperature, indicating that the energy loss of the internal system of the material decreases at high temperatures. At this time, the mechanical properties and stability are improved macroscopically.

Data pertaining to the storage modulus and loss factor of BOPA6 with different stretching ratios are shown in Figure 9b,e. Compared with sample B-1515, the loss modulus of sample B-33 is increased by 500 MPa, consistent with the trend observed under different stretching temperature conditions. The reason is that the high stretch ratio means the molecular chains are stretched longer, and the orientation is increased, thereby enhancing the stiffness of BOPA6 and increasing the storage modulus. In DMA testing, the glass transition temperature (*T_g_*) is defined as the temperature at which the peak of the loss factor (Tan δ) curve occurs [31]. The temperature at which the peak of the loss factor curve occurs is 60 degrees Celsius for stretch ratios of 1.5 × 1.5 and 2 × 2, which is close to the glass transition temperature of the PA6 material. However, for stretch ratios of 2.5 × 2.5 and 3 × 3, the temperature at which the peak of the Tan δ curve occurs is 90 °C, indicating that the *T_g_* of the material increases and the thermal stability is better at a high stretching ratio.

DMA results of BOPA6 at different stretching rates are shown in Figure 9c,f. BOPA6 molecular chains have more time to rearrange and form crystalline regions at low stretching rates, resulting in a higher storage modulus of BOPA6. In addition, it can be observed that under the same stretching temperature and ratio, the storage modulus and loss factor of BOPA6 film show little variation with different stretching rates, and the curves overlap closely, indicating that different stretching rates have little impact on the thermal stability of BOPA6.

**Figure 10:**
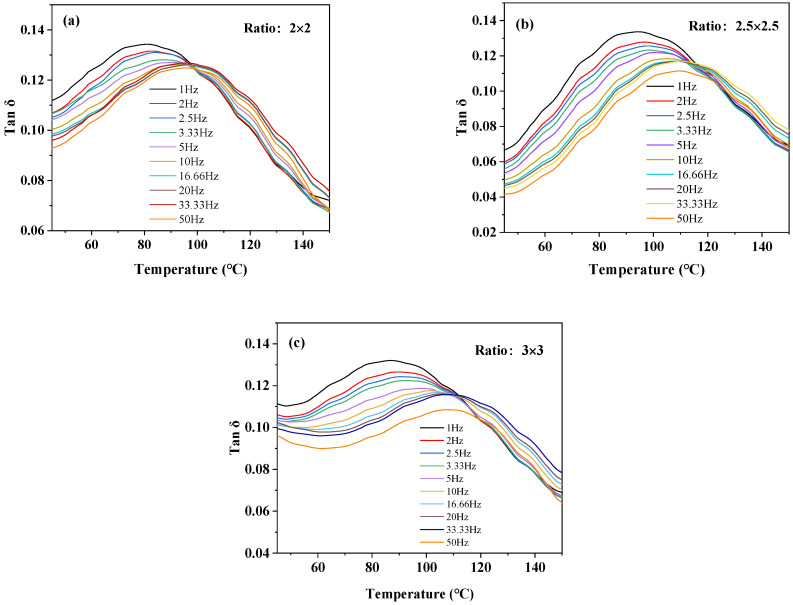
Multi-frequency temperature scan DMA curves of BOPA6 films with different stretching ratios: (**a**) stretching ratio, (**b**) stretching ratio 2.5 × 2.5, (**c**) stretching ratio 3 × 3.

Figure 10 shows the multi-frequency temperature scan DMA curves of BOPA6 film with different draw ratios, and it can be seen that the temperature at the peak of the loss factor curve shifts to high temperatures as the frequency increases under different stretching ratio processes. Table 6 shows the corresponding *T_g_* of BOPA6 film with different stretching ratios at different frequencies.

**Figure 11:**
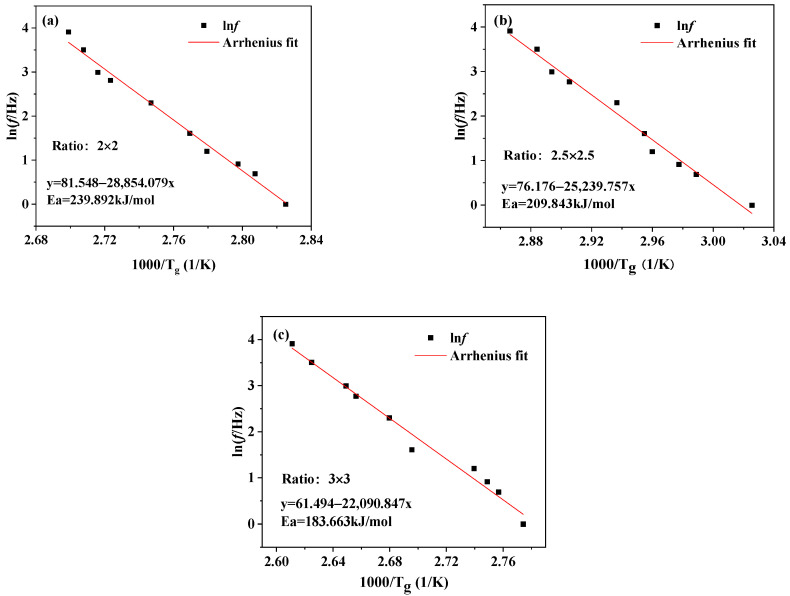
Arrhenius fitting curves of BOPA6 films at different stretching ratios: (**a**) 2 × 2 stretching ratio, (**b**) 2.5 × 2.5 stretching ratio, (**c**) 3 × 3 stretching ratio.

According to Equation (4), the logarithm of frequency is linear with the reciprocal of glass transition temperature. Using Equation (4) to fit the data in Table 6, the activation energies (E_a_) of BOPA6 with different stretching ratios can be calculated as E_a2_ = 239.892 kJ/mol, E_a2.5_ = 209.843 kJ/mol, E_a3_ = 183.663 kJ/mol, respectively, as shown in Figure 11. The results show that the *E_a_* decreased by 56.229 kJ/mol when the stretching ratio changed from 2 × 2 to 3 × 3; this can be attributed to the BOPA6 molecular chain being stretched and oriented during the stretching process, resulting in the internal movement and re-arrangement of the molecules. From the perspective of BOPA6 crystallization, because the increase of the stretching ratio will increase the crystallinity, the formation of crystallization can reduce the randomness of molecular chain movement, the molecular chain more easily responds to external stress and deforms, changes the distance and interaction mode between the original molecules, thus reducing the E_a_.

## 4. Conclusions

In this paper, the biaxially-oriented polyamide 6 (BOPA6) film was used as the research object, and the rheological properties of PA6 were investigated first. PA6 cast film was obtained by extrusion casting method, and then BOPA6 film was prepared by synchronous biaxial stretching method. The effects of different biaxial stretching processes on the crystallization behavior, barrier, and optical and mechanical properties of BOPA6 were investigated, and the following conclusions were obtained:

(1) The change in temperature has an important influence on the physical properties of BOPA6. The increase in stretching temperature is beneficial to the flow and directional arrangement of BOPA6 molecular chains, which makes the material more prone to deformation and reduces the stretching force required for the biaxial stretching process; the force required for the biaxial stretching is 45N at 180 °C, which is 55% lower than that at 70 °C. In addition, increasing the biaxial stretching temperature can promote the crystallization of BOPA6, improve its tensile strain and barrier properties, and reduce the haze. The tensile strength is relatively stable at high stretching temperatures, reaching peak values at 120 °C; the tensile strength of MD and TD were 232% and 270%, respectively, indicating that at this temperature, the material had good toughness and could withstand large pre-fracture deformation. When the temperature is above the glass transition temperature and below the melting point, the storage modulus of the BOPA6 film increases with the increase of the stretching temperature and the loss factor decreases;
(2) At the same stretching temperature and rate, the force required for the biaxial stretching process increases with the increase of the stretching ratio. When the stretching ratio is 3 × 3, the force required to complete the stretching is 85 N. At a large stretching ratio, the stress concentration in the TD direction increases, which affects the force distribution in the biaxial stretching process. Through the DSC and XRD data, it was found that strain-induced crystallization (SIC) occurs during the biaxial stretching process with different stretching ratios. The formation of crystallization reduced the randomness of molecular chain movement and reduced the activation energy. The grain size reached its maximum value at a stretching ratio of 2.5 × 2.5, which was 37 Å, but excessive stretching ratios can lead to a decrease in grain size. When the stretching ratio is 3 × 3, the melting enthalpy is 69.785 J/g, which increased by 32.39% compared with the unstretched sample. With the increase of the stretching ratio, the structure of BOPA6 film becomes more uniform and compact, the haze is significantly reduced, decreases by 4.2% compared to the unstretched sample, and the stretching strength and oxygen barrier properties are improved. When the tensile ratio was 3 × 3, it reached about 150 MPa, which was twice the tensile strength of the unstretched sample;
(3) Changing the stretching rate can adjust the force of the biaxial stretching process. It can be observed that when the stretching rate increases from 25%/s to 50%/s, the force decreases from 85 N to 71 N. However, at higher stretching rates (100%/s, 200%/s, 300%/s), the force required for biaxial stretching is approximately the same, about 64N. The grain size reached its maximum value of 39 Å at a stretching rate of 100%/s, indicating that there is an optimal stretching rate that promotes grain growth up to a certain extent. When the crystals in the BOPA6 film are influenced by high strain rates, this leads to an increase in the degree of crystal defects and disorder. With the increase of the stretching rate, the haze of BOPA6 increases and the oxygen barrier property decreases, the tensile strength in the MD direction is always higher than that in the TD direction, and the overall trend of failure strain during tensile deformation shows a decrease.

## Figures and Tables

**Table 1:** Temperature zone parameters of the extruder casting machine.

Sample	Feed Cylinder (°C)				Transition Section (°C)		Mold Head (°C)			Mold Lip (°C)
	Zone 1	Zone 2	Zone 3	Zone 4	Zone 1	Zone 2	Zone 1	Zone 2	Zone 3	Zone 1
PA6	215	250	250	255	255	255	255	255	255	250

**Table 2:** Biaxial stretching process.

Biaxial Stretching Process	Sample	Preheating Time	Stretching Temperature	Stretching Ratio	Stretching Rate	Setting Temperature	Setting Time
		(s)	(°C)	(MD × TD)	(%/s)	(°C)	(s)
Different stretching temperatures	W-70	30	70	2 × 2	100	100	60
	W-90	30	90	2 × 2	100	120	60
	W-120	30	120	2 × 2	100	150	60
	W-160	30	160	2 × 2	100	190	60
	W-180	30	180	2 × 2	100	210	60
Different stretching ratios	B-11	30	160	1 × 1	100	190	60
	B-1515	30	160	1.5 × 1.5	100	190	60
	B-22	30	160	2 × 2	100	190	60
	B-2525	30	160	2.5 × 2.5	100	190	60
	B-33	30	160	3 × 3	100	190	60
Different stretching rates	S-25	30	160	2.5 × 2.5	25	190	60
	S-50	30	160	2.5 × 2.5	50	190	60
	S-100	30	160	2.5 × 2.5	100	190	60
	S-200	30	160	2.5 × 2.5	200	190	60
	S-300	30	160	2.5 × 2.5	300	190	60

**Table 3:** Dimensions of the mechanical tensile specimens.

Gauge Length (L_0_)	Narrow Portion Width (B_1_)	Initial Distance between Fixtures (L)	Narrow Part Parallel Length (L_1_)	Overall Length (L_3_)
20 mm	4 mm	50 mm	30 mm	75 mm

**Table 4:** Crystallization and thermal properties data of BOPA6 under different stretching processes.

Biaxial Stretching Process	Sample	*T*_m_	Δ*H_m_*	Crystallinity	FWHM	Grain Size
		(°C)	(J/g)	(%)		(Å)
Different Stretching temperature	W-70	221.97	62.611	27.22	3.629	22
	W-90	222.01	63.021	27.40	3.559	22
	W-120	222.05	63.421	27.57	3.136	43
	W-160	222.06	63.465	27.59	2.247	26
	W-180	222.11	64.119	27.97	2.089	35
Different Stretching ratio	B-11	222.87	60.260	26.20	5.385	15
	B-1515	222.56	63.076	27.42	3.479	23
	B-22	222.63	63.234	27.49	2.422	33
	B-2525	221.96	66.295	28.82	2.214	37
	B-33	221.50	69.785	30.34	2.324	35
Different Stretching rate	S-25	221.47	63.708	27.70	2.214	37
	S-50	221.42	60.925	26.49	2.113	38
	S-100	221.32	59.477	25.86	2.085	39
	S-200	221.54	51.809	22.53	2.198	37
	S-300	221.50	49.919	21.70	2.323	35

**Table 5:** Oxygen permeation test results of BOPA6 under different stretching processes.

Biaxial Stretching Process	Sample	Transmission Rate	Permeation
		cc/(m^2^·Day)	cc·mil/(m^2^·Day)
Different stretching temperatures	W-140	25.623	70.617
	W-160	31.761	69.538
	W-180	35.326	66.273
Different stretching ratios	B-22	26.184	79.377
	B-2525	27.171	53.448
	B-33	38.505	57.606
Different stretching rates	S-100	22.662	47.286
	S-200	29.808	50.463
	S-300	32.080	59.361

**Table 6:** *T_g_* of BOPA6 film at different frequencies under different stretching ratio processes.

*f*/Hz		1	2	2.5	3.33	5	10	16.66	20	33.33	50
2 × 2	*T_g_*/K	353.96	356.21	357.47	359.80	361.09	364.05	367.20	368.20	369.33	370.52
2.5 × 2.5		330.52	334.59	335.86	337.86	338.44	340.53	344.19	345.57	346.74	348.89
3 × 3		360.45	362.73	363.79	365.03	370.98	373.17	376.48	377.48	380.98	382.90

## Data Availability

Data are contained within the article.
